# Age structure of amphibian populations with endemic chytridiomycosis, across climatic regions with markedly different infection risk

**DOI:** 10.1002/ece3.9123

**Published:** 2022-07-24

**Authors:** Anna Turner, Geoffrey Heard, Andrew Hall, Skye Wassens

**Affiliations:** ^1^ School of Agricultural, Environmental and Veterinary Science Charles Sturt University Albury New South Wales Australia; ^2^ Terrestrial Ecosystem Research Network The University of Queensland Indooroopily Queensland Australia

**Keywords:** amphibian declines, conservation, demography, growth curves, life‐history

## Abstract

Threatening processes, such as disease, can drive major changes in population demographics of the host. Chytridiomycosis, caused by the fungal pathogen *Batrachochytrium dendrobatidis* (*Bd*), has led to the decline of at least 500 amphibian species across the globe and has been shown to truncate host age structure by lowering adult survival rates. This results in heavy reliance on annual recruitment and the inability to recover in the event of periodic recruitment failure. We used skeletochronology to determine the age structure, growth, and survival rates of populations of an endangered amphibian, *Litoria raniformis*, with endemic chytridiomycosis, across two climatically disparate regions in south‐eastern Australia: semi‐arid and temperate. Contrary to predictions, populations in the semi‐arid region (in which chytrid prevalence is substantially lower due to high temperatures) displayed a more truncated age structure than populations in the temperate study regions. Maximum recorded age was only two years in the semi‐arid region compared with up to four years in the temperate region. Wetland hydroperiod and average seasonal air temperature were correlated with age, and males had a slightly higher survival rate than females (0.31 for males and 0.27 for females). Despite the previously documented differences in chytrid prevalence between the two climatic regions, water availability and wetland hydroperiods appear the over‐riding determinants of the age structure and survival rates of *L. raniformis*. Targeted management which ensures water availability and improves survival of 1‐year‐old frogs into their second and third breeding season would reduce the impact of stochastic events on *L. raniformis*, and this may be true for numerous frog species susceptible to chytridiomycosis.

## Graphical abstract

Populations of a chytrid‐infected amphibians, *Litoria raniformis*, are dominated by relatively young individuals at both the semi‐arid and temperate extent of their range. Hydroperiod and average seasonal air temperature are key environmental drivers of age and survival rates.

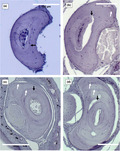

## 1 INTRODUCTION

Amphibian populations are in decline globally and are considered the most threatened group of vertebrates (Scheele et al., 2019; Wake & Vredenburg, 2008). When populations are under threat, an understanding of their demography and the factors influencing survival rates can help target conservation efforts (Muths et al., 2018). Population age structure and survival rates in amphibians are driven by a number of factors, including extent and continuity of suitable breeding habitat (McCaffery et al., 2014), water quality (Bounas et al., 2020), presence of invasive species (Falaschi et al., 2020), and disease (Campbell et al., 2018; McCaffery et al., 2015). The age structure of a population can provide insights into the potential for population size fluctuations (Sinsch, 2015). In stable populations, where recruitment and mortality are consistent, it is expected that the number of individuals will gradually reduce over successive age classes from youngest to oldest (Biek et al., 2002; Middleton & Green, 2015). Sporadic recruitment or mortality can lead to variability in age structure and consequently an unstable population (McCaffery et al., 2014, 2015).

Populations of short‐lived individuals are especially susceptible to extinction when recruitment failure occurs (Angelini et al., 2018; Biek et al., 2002). High rates of adult mortality in amphibian populations suffering from chytridiomycosis, the disease caused by aquatic fungus *Batrachochytrium dendrobatidis* (*Bd*) (Berger et al., 1998), can lead to a truncation of age structure, reducing the number of individuals capable of reproducing (Campbell et al., 2018). Chytrid‐infected amphibian populations therefore rely heavily on consistent annual recruitment, and, in the event of periodic recruitment failure, any population with a truncated age structure is often unable to recover (Scheele et al., 2016).

The endangered southern bell frog, *Litoria raniformis*, is an Australian hylid species that has suffered population declines due to *Bd* (Heard et al., 2012), in combination with other stressors such as habitat loss and fragmentation and changes to flooding regimes (Wassens et al., 2010). Previous research using mark‐recapture has demonstrated *L. raniformis* displays rapid growth and maturation and suggests that populations with endemic chytridiomycosis display low adult survival rates with very few animals likely to live beyond their first breeding season (Heard et al., 2012). However, survival rates for *L. raniformis* may be generally higher than those estimated by Heard et al. (2012), as their mark‐recapture data did not separate mortality from permanent emigration from the study area.

Amphibian growth is often rapid until sexual maturity, then slows, making size a poor indicator of age (Sinsch, 2015). For species with clear annual cycles of activity and growth, skeletochronology may be used to construct age structures, estimate annual survival rates, and derive growth curves (McCreary et al., 2008; Sinsch & Aguilar‐Puntriano, 2021). Skeletochronology is based on the observation of lines of arrested growth (LAGs) in a cross section of bone, taken non‐lethally from a phalange clipped from a captured individual (McCreary et al., 2008; Székely et al., 2018).

Successful aging of *L. raniformis* has been achieved using skeletochronology for populations in Tasmania (Ashworth, 1998) and in the semi‐arid Coleambally region in south‐western New South Wales (Mann et al., 2010). In the latter study, laboratory‐reared animals of known ages were used to test the method and displayed clear corresponding LAGs corresponding to over‐wintering periods (Mann et al., 2010). Results suggested survival rates in semi‐arid New South Wales may be higher than those in temperate regions as estimated by Heard et al. (2012). If so, this disparity in survival rates may stem from differing levels of exposure to chytridiomycosis. It is plausible that the demographic impact of chytridiomycosis varies markedly across the range for *L. raniformis*, with the geographic distribution of this species spanning from cool temperate in the south of its range to semi‐arid in the north (Pyke, 2002; Schultz, 2008; Wassens, 2008).

In a previous study (Turner, Wassens, & Heard, 2021), we showed that the prevalence and intensity of *Bd* infections among *L. raniformis* differs markedly between semi‐arid and temperate portions of the range of *L. raniformis*. In the semi‐arid zone in which maximum temperatures during the active season are 4.5°C higher on average and rainfall is 50% lower, observed seasonal *Bd* prevalence is 8% compared with 23% in the temperate region. Patterns of infection intensity also followed this pattern, with zoospore load among infected individuals being 77% higher on average in the temperate region. With *Bd* infections being an important source of mortality for *L. raniformis* (Heard et al., 2014), it is reasonable to hypothesize that impacts on demography will be apparent; in particular, greater truncation of age structures in temperate regions due to higher mortality from *Bd*.

**FIGURE 1:**
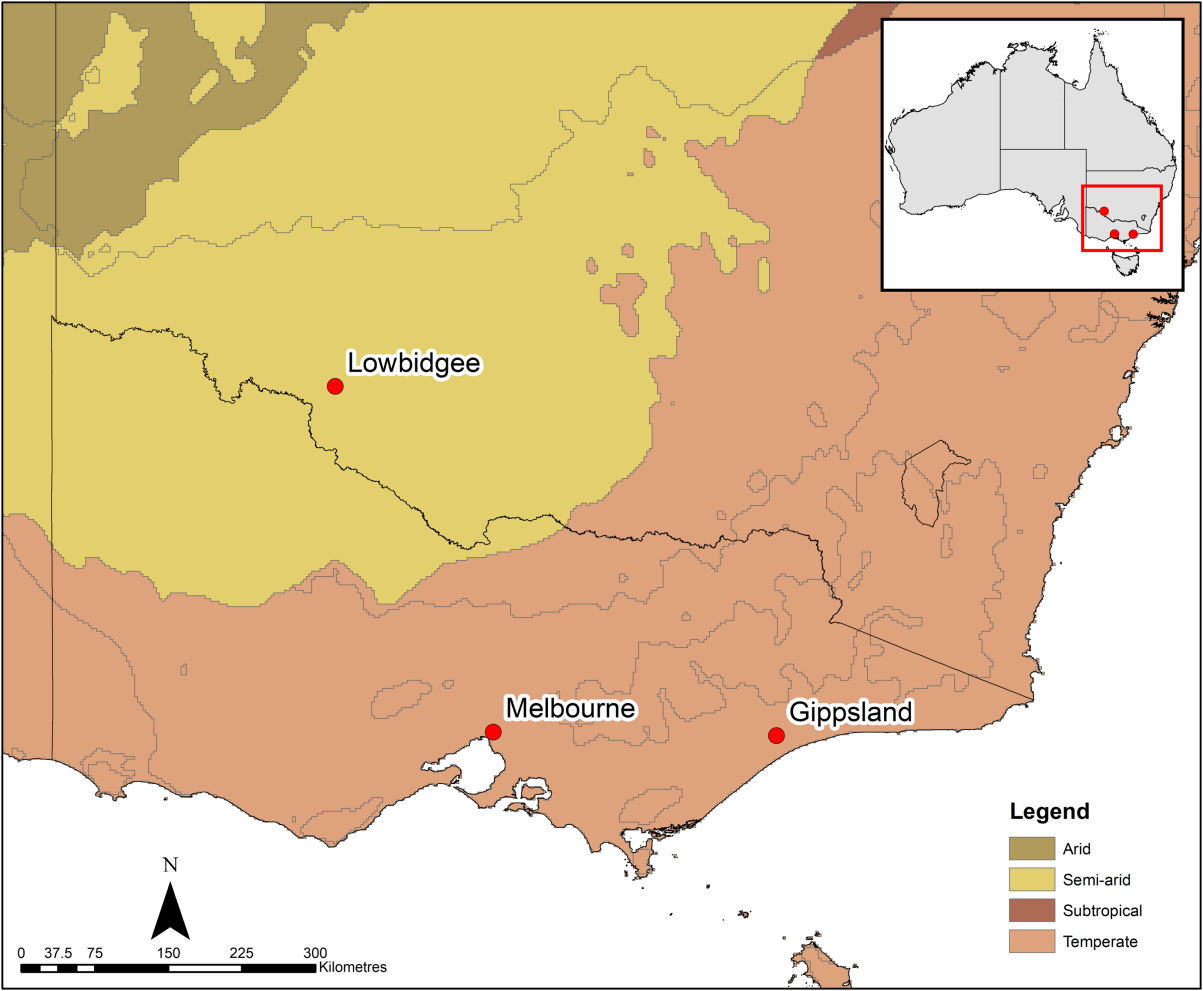
Location and climate classification of the three study regions in South‐Eastern Australia, the semi‐arid (Lowbidgee) and temperate (Melbourne and Gippsland) regions.

Here, we assessed differences in the demography of *L. raniformis* between these climatically disparate portions of the species' range, with semi‐arid populations being represented by those in the ‘Lowbidgee’ area of south‐western New South Wales and temperate populations represented by those in Melbourne and Gippsland in Victoria (Figure 1). These are the same populations studied by Turner, Wassens, Heard, and Peters (2021), in which the significant differences in infection risk were detected. We asked four specific questions: asking four specific questions:

1. How does the age structure of *L. raniformis* populations differ between temperate and semi‐arid regions, in which *Bd* infection risk disease risk is substantially different?
2. Within regions, what additional environmental variables are correlated with age structure?
3. Do growth rates of *L. raniformis* differ between temperate and semi‐arid regions?
4. What factors influence survival rates of *L. raniformis*?

## 2 METHODS

### 2.1 Study species

*L. raniformis* is considered a habitat generalist, found to occupy permanent lakes, dams, streams, and seasonally flooded wetlands (Anstis, 2013). Historically, *L. raniformis* was widespread and locally abundant through southern New South Wales (NSW), the Australian Capital Territory (ACT), eastern South Australia and Victoria on the mainland, as well as King Island, Flinders Island and northern and eastern Tasmania (Pyke, 2002; Wassens, 2008).

The abundance and geographic range of *L. raniformis* declined rapidly in the south eastern highlands of NSW and the ACT in the late 1970's and early 1980's (Osborne et al., 1996). Similar declines in populations of *L. raniformis* occurred prior to 1989 in the western division of NSW, central Victoria and parts of Tasmania and South Australia (Mann et al., 2010; Pyke, 2002; Schultz, 2008; Wassens, 2008). Declines have occurred due to habitat loss and fragmentation, predation by invasive fish, drought and changes in flooding regimes and *Bd*, with the precipitous declines of the late 1980s and early 1990s being driven by *Bd* in particular (Clemann & Gillespie, 2012; Wassens, 2008). Current populations exist in strongholds in the lower Murrumbidgee, lower Murray, and parts of southern Victoria, South Australia, and Tasmania.

### 2.2 Location and climate of study regions

Here, we incorporate data collected from two climatically distinct regions in south‐eastern Australia (Figure 1). A total of seventy surveys were conducted across fifteen sites in the semi‐arid environs of the Lower Murrumbidgee floodplain (‘Lowbidgee’) in south‐western New South Wales (closest town: Hay, 34.504°S, 144.844°E) during the 2018/2019 breeding season of *L. raniformis* (September–March). In the temperate Gippsland region of Victoria (closest town: Sale, 38.102°S, 147.073°E), fifty‐four surveys were conducted across fifteen sites during the 2018/2019 breeding season. Surveys in the Melbourne region (Merri Creek: 37.761°S, 144.983°E) were conducted during the 2004/2005 and 2005/2006 breeding seasons, covering 17 sites in the first season and seven in the second (123 surveys in total; see Heard et al., 2012, 2014). Data from Melbourne surveys were combined with that from Gippsland to represent temperate populations, given limited captures in Gippsland and the climatic similarities of these regions.

Average seasonal temperature (1990–2020), in each town closest to the survey points highlights the climatic disparity between regions (BOM., 2021; Murray et al., 2010). The mean maximum summer temperature in the semi‐arid region is 32.4°C, which is near 7°C warmer than the temperate region (25.7°C). Autumn mean maximum temperature in the semi‐arid region (24.3°C) is 3°C warmer than the temperate region (20.8°C), and spring temperatures are 3.5°C degrees higher in the semi‐arid region (semi‐arid = 24.8°C, temperate = 20.6°C). Melbourne and Gippsland have on average double the annual rainfall of the Lowbidgee (640 mm/yr compared to 323 mm/yr; BOM, 2021).

### 2.3 Frog surveys and tissue sampling

Spotlight surveys were conducted after dark, along the water's edge of streams, farm dams, swamps, and flooded quarries. Frogs were captured by gloved (nitrile glove) hand or small hand‐held net. Snout‐urostyle length (SUL) of each captured individual was recorded, and frogs were weighed (0.01 g precision) using a spring balance (Pesola Micro‐Line Spring scale, 100 g, Pesola Präzisionswaagen AG, Switzerland). Males were identified by the presence of nuptial pads and throat colouration of yellow, brown, or black (Ashworth, 1998; Hamer & Mahony, 2007; Heard et al., 2012). Adult females lacked these secondary sexual characteristics; their throat is white and their body size generally larger than adult males.

The third digit on the left forehand was clipped for the purposes of skeletochronology. Standard procedures were followed (DECC, 2008; Mann et al., 2010; McCreary et al., 2008). Wounds were treated with Bactine® (Bayer, Morristown, New Jersey, USA)—a topical antiseptic and anesthetic—and the wound sealed with the surgical glue Vetbond® (3 M Animal Care Products, St. Paul, Minnesota, USA). The tissue and bone sample were stored in a 5 ml vial of 95% ethanol. In the Lowbidgee, 184 toe clips were obtained, 11 from Gippsland, and 393 from Melbourne. As above, due to the low number of samples collected from Gippsland, data were combined with that from Melbourne to represent the temperate population.

### 2.4 Environmental variables measured at survey sites

A suite of environmental variables were recorded at each study site to explore whether local‐scale drivers may be important additional factors determining the demography of *L. raniformis*. Site hydroperiod was scored as the period of inundation, using the following ordinal scale: ‘Ephemeral’ (1), ‘Semi‐permanent’ (2), and ‘Permanent’ (3). Ephemeral sites were those considered to fill intermittently, retaining water for only months at a time after filling events. Semi‐permanent sites were those that retain water in most years but are dry during low rainfall periods. Permanent sites were those that always hold water, regardless of climatic fluctuations. Percentage emergent vegetation in the survey transect was visually assessed according to Sainty and Jacobs (2003). Water chemistry parameters (temperature [°C], pH, conductivity [mScm^−1^]) were measured using hand‐held meters (Horiba U‐52 Multiparameter meter, Horiba, Ltd. Kyoto, Kyoto, Japan or Oakton CON11 water quality meter, Oakton Instruments, Vernon Hills, Illinois, USA).Temperature loggers deployed at each site were used to calculate average seasonal air and water temperatures along with proportion of air and water temperatures within the chytrid thermal optimum (17–26°C) (Turner, Wassens, & Heard, 2021).

### 2.5 Age determination via skeletochronology

Bone specimens were processed using conventional histological methods involving paraffin embedding, sectioning, and staining (McCreary et al., 2008). Melbourne samples were processed following Scheele et al. (2015), Lowbidgee and Gippsland samples were processed following McCreary et al. (2008). Differences in the methods were slight changes to decalcification and staining times and had negligible effects on the outcomes.

**FIGURE 2:**
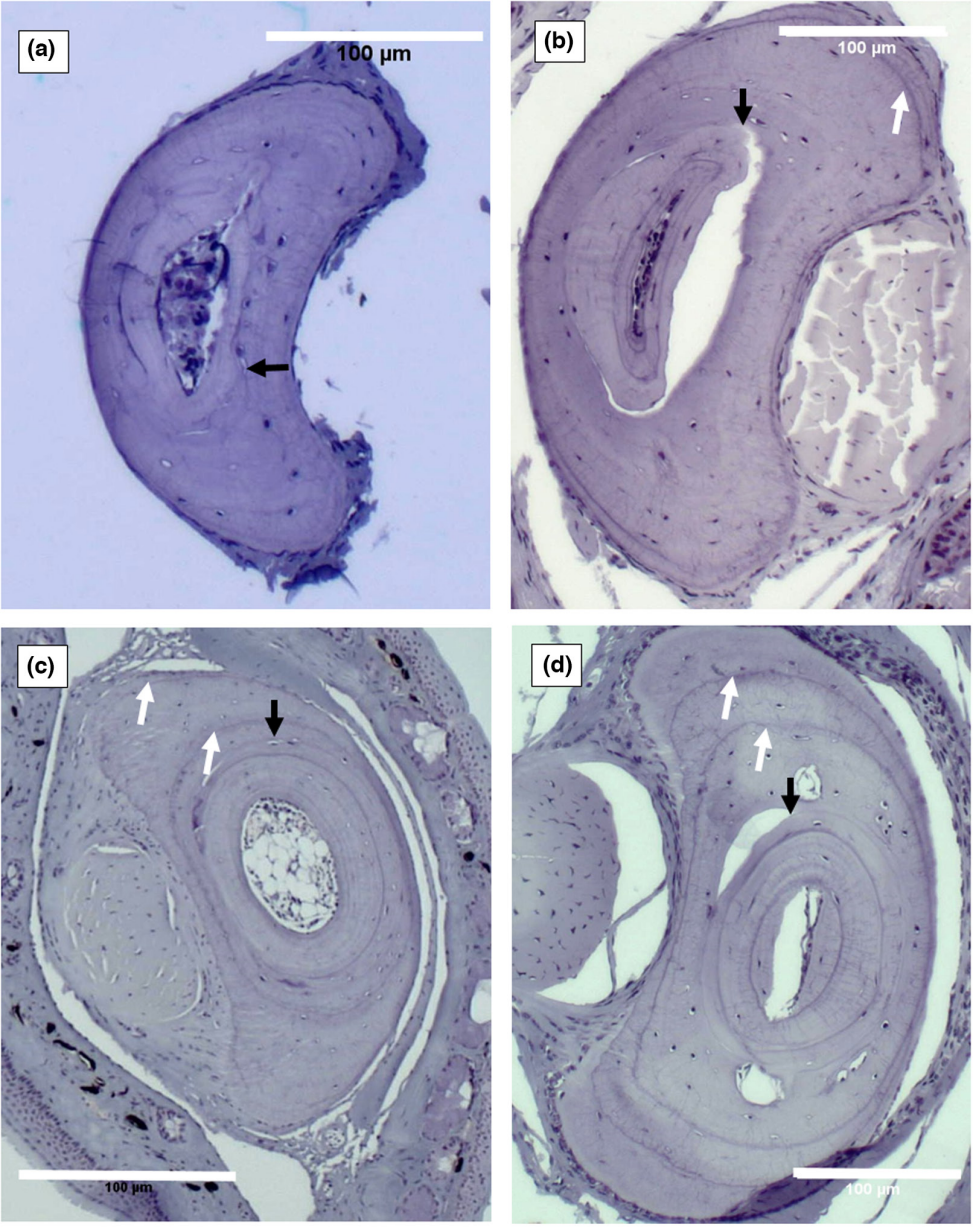
Histological sections of toe‐bones of *Litoria raniformis*. In each micrograph, thick black arrows indicate the line of metamorphosis (the limit of bone resorption) separating the inner endosteal bone and outer periosteal bone. White arrows indicate lines of arrested growth (LAG). (a) Young of year with SUL of 46.8 mm, caught 24th January 2019 at nap nap, Lowbidgee; (b) one LAG, SUL of 66.5 mm, caught 25th November 2018 at pollen dam, Lowbidgee; (c) two LAGS, male, SUL of 79 mm 4th Feb 2019 Crombe wetland, Gippsland; (d) two LAGs, male, SUL 64.1 mm, caught 4th Feb 2019 Crombe wetland, Gippsland. The scale bar in each micrograph is 100 μm data analysis.

Each line of stain visible in the bone cortex was taken to represent one period of arrested growth (LAG) (Figure 2) (Mann et al., 2010). Timing of capture and body size were used to guide aging when LAGs were faint or ambiguous. Individuals of any size captured in September, October, or November with a weak or partial LAG could be confirmed to have passed through one winter, as overwintering of tadpoles has not been observed in the field (A. Turner, G. Heard, S. Wassens pers. obs.) and is very unlikely given their sensitivity to low water temperatures (Cree, 1984). As such, even individuals at metamorph size with a weak or partial LAG that were captured in Spring could confidently be assigned to one‐year old. Similarly, individuals with no obvious LAGs captured in November–April (semi‐arid sites) or December–April (temperate sites) with SUL <50 mm were assumed to be young‐of‐the‐year (YOTY). The reasoning was as follows: (i) First, metamorphs were detected from November (semi‐arid) or December (temperate) both visually and through high‐quality skeletochronology samples; (ii) the rapid growth rates of *L. raniformis* (Heard et al., 2012) ensure that one‐year‐old individuals would be larger than 50 mm SUL by that point in the season. Nevertheless, caution was applied, and a conservative approach was taken where ambiguity remained. For example, several individuals from Lowbidgee captured late in the season with SULs just above 50 mm and with no obvious LAGs were not aged due to poor quality sections, despite captures of zero‐aged individuals of the same size captured at the same site on the same date. After discarding unreliable sections (those scored as ‘discard’ or ‘low’), 82 remained from Lowbidgee, 9 from Gippsland, and 296 from Melbourne. A selection of slides is presented here (Figure 2) as examples of the skeletochronology results and LAG readings obtained in the study. In total, 387 skeletochronological readings were available for analysis.

To supplement the skeletochronology data, individuals who could be confidently assigned to young‐of‐the‐year (YOTY) that were captured during surveys but not toe‐clipped were also compiled. Doing so was necessitated by the underrepresentation of YOTY in the skeletochronology data relative to their true abundance, due to large numbers of YOTY encountered during some surveys and the inability to include all these individuals in skeletochronological assessments for both logistical and financial reasons. The rules above were followed to identify YOTY that were not bone‐sampled. A total of 73 YOTY were added from semi‐arid sites and 307 from temperate sites.

#### 2.5.1 Development of candidate model set for age structure

Despite the extensive *Bd* prevalence and intensity data collected across these sites by Turner, Wassens, Heard, and Peters (2021), robust and comparable estimates of these parameters site‐by‐site were not available due to variation in capture and sampling success. As such, site‐level estimates of *Bd* infection risk could not be included as predictors in models of the age structure of *L. raniformis*, with focus instead on regional differences corresponding to documented variation in *Bd* infection prevalence and intensity (as reported by Turner, Wassens, Heard, & Peters, 2021).

Ten additional environmental variables were identified as having plausible mechanisms linking them to survival rates and age structure of *L. raniformis* in both climatic regions, either directly or through their effect on chytrid prevalence and intensity (Table 1). Correlations between variables were tested first, with pairs of variables with correlation coefficients >0.5 excluded from the same model. Due to high correlations between temperature variables, only the following were included: (i) the average night‐time air and water temperature across the active season and (ii) the proportion of night‐time air and water temperature recordings across the primary active season in the range 17 to 26°C (the optimal range for growth of chytrid zoospores). Night‐time temperatures were defined as the mean of the eight‐hour period centre on solar midnight (between 21:00 and 05:00 Australian Eastern Daylight‐Saving time), and the primary active season as beginning of October to end of March.

**TABLE 1:** Environmental variables considered potential determinants of age of *Litoria raniformis* at semi‐arid and temperate sites. Variable selection and expected relationships are based on mechanisms linking them to the prevalence and intensity of *Bd* identified in Turner, Wassens, and Heard (2021) along survival and growth of *Bd* in vitro, in vivo (in laboratory experiments) and in situ (in the field) identified in the literature and the possible impact on age of individual frogs (see ‘source’).

Variable	Relationship/s	Mechanism (s)	Source
Lagged temperature variables			
Proportion of night‐time water/air temperature readings between 17 and 26°C during springs Proportion of night‐time water/air temperature readings between 17 and 26°C during summer Proportion of night‐time water/air temperature readings between 17 and 26°C during frog active season spring–autumn Average night‐time water/air temperature in spring Average night‐time water/air temperature in summer Average night‐time water/air temperature during frog active season	Negative linear	Within the specified season, as the proportion of night‐time temperature, in the optimum growth range for *Bd* increases, *Bd* prevalence will increase and therefore decrease annual survival rates of *L. raniformis*	Longcore et al. (1999); Murray et al. (2009); Whitfield et al. (2012)
*Vegetation parameters*			
Emergent vegetation cover (%)	Positive linear	As percentage of emergent vegetation increases, waterbodies potentially become more thermally stable within the optimum growth range for *Bd*. This could lead to higher infection rates and decreased survival	Clemann et al. (2013); Heard et al. (2014); Heard et al. (2015)
	Negative linear	However, emergent vegetation provides shelter from predators, calling platforms for breeding, substrate to attach frog spawn, food for tadpoles, diversity for insects, and other species as food source, which could increase survival rates	
Salinity (as electrical conductivity, μS/cm)	Positive linear	A negative linear relationship was detected between salinity and chytrid prevalence in Turner, Wassens, and Heard (2021) as *Bd* is intolerant of saline conditions. Therefore, increasing salinity will decrease prevalence of infections. This in turn will increase chances of survival and older frogs detected in the population	Heard et al. (2014); Stockwell et al. (2012, 2014)
	Quadratic effect	A quadratic effect of salinity could also be possible due to extremely high salinity recordings in the Gippsland sites. Very high levels of salinity are not tolerated by amphibians, leading to a decrease in survivability	
pH	Quadratic	Chytrid is sensitive to pH, displaying markedly higher growth and survival in a narrow band of slightly acidic to neutral conditions (pH of 6–7). We detected a quadratic effect of pH on chytrid prevalence. Infection prevalence and intensity may be higher within this band, decreasing survival and age of individual frogs	Piotrowski et al. (2004)
Hydroperiod^a^	Positive linear	As *Bd* is an aquatic fungus and is sensitive to desiccation, waterbodies that dry out for a period may have reduced levels of *Bd* However, frogs also need a certain length hydroperiod to complete metamorphosis, an intermittent hydroperiod may mean frogs only breed in certain years, reducing the survival rate	Murphy et al. (2011); Stockwell et al. (2012)

^a^ Hydroperiod was scored as the frequency of inundation, using the following ordinal scale: ‘Intermittent’ (1), ‘Ephemeral’ (2), ‘Semi‐permanent’ (3) and ‘Permanent’ (4).

Nineteen candidate models were developed in total (Table S1), guided by: (1) temperature, pH, and salinity as drivers of *Bd* infection prevalence and intensity identified in Turner, Wassens, and Heard (2021) and (2) other potential drivers of survival and age of amphibians such as hydroperiod and aquatic vegetation (Wassens et al., 2010). Despite predicted effect of sex on survival rate and therefore age structures, sex was not included in the model set as it was unknown for immature individuals.

#### 2.5.2 Model fitting for age structure

Only frogs with a skeletochronology reading of medium or high reliability were used in the analysis. Salinity (conductivity) data were log transformed prior to analysis. All variables were standardized by subtracting the mean and dividing by two standard deviations.

The candidate model set was fitted to the full age structure data to determine relationships between age of *L. raniformis* and the environmental covariates listed in Table 1. Generalized linear mixed effects models with a Poisson distribution were fitted to the age structure data with the aid of LME4 package in R version 4.0.3 (R Development Core Team, 2021). ‘Climate’ (as a categorical variable, either semi‐arid or temperature) was included as a random effect in all models to estimate the systematic differences in age structure hypothesized between semi‐arid and temperate regions, due to differences in chytrid infection risk. ‘Site’ was also included as a random effect to allow frogs from the same site to have correlated age structures. Models were ranked using Akaike Information Criterion (AIC; Burnham & Anderson, 2002).

#### 2.5.3 Growth curves

Post‐metamorphic growth rates of male and female *L. raniformis* were estimated by fitting the von Bertalanffy model (Von Bertalanffy, 1938) to size‐at‐age data using Bayesian non‐linear regression with Markov Chain Monte Carlo (MCMC) sampling in JAGS (Plummer, 2003) called from R via the R2jags package (Su et al., 2015). The von Bertalanffy model has been successfully used for post‐metamorphic growth rates of numerous amphibians (Gibbons & McCarthy, 1984; Heard et al., 2012; Hemelaar, 1988; Miaud et al., 2000). The classical ‘size‐at‐age’ version of this model predicts body size (SUL) at a given age, *t*, as:

$$\mathrm{SUL}=\alpha \left[1-\beta \left(e^{-\lambda t}\right)\right],$$

where α is the asymptotic body size, β is the fraction of the asymptotic body size yet to be attained at birth (where ‘birth’ is metamorphosis and metamorph body size was set to 34.3 following Heard et al., 2012), and λ is the growth coefficient (i.e., shape of growth curve). Only individuals whose sex could be reliably determined were included in the analysis. Estimates of the parameters of the von Bertalanffy model were obtained from 30,000 MCMC samples after discarding the first 30,000 samples as a burn‐in.

Frogs with zero LAGs that were >40 mm were excluded from the data as their age could have ranged from weeks to many months of age, and their inclusion led to poor model fit. For each sex, asymptotic size and growth rate were allowed to vary between semi‐arid and temperate regions by drawing both parameters from a normal distribution with a mean defined by the estimates of these parameters from Heard et al. (2012), and a standard deviation to be estimated from the data. Growth curves were subsequently estimated for semi‐arid and temperate sites for both sexes.

#### 2.5.4 Survival rates

Annual survival rates of adult *L. raniformis* were estimated from the age structure data using the ‘catch‐curve’ approach of Scroggie (2012). Under the assumption that all adult age classes experience equivalent survival rates, age structure data are decreasing geometric series (Seber, 1986) from which the interval survival rate may be estimated (Chapman & Robson, 1960; Jensen, 1985). The approach has been widely used in fisheries research, with several applications to amphibians aged by skeletochronology (Lee et al., 2010; Miaud et al., 2000). The approach of Scroggie (2012) allows survival rate to be modeled as a function of covariates using a linear equation and logistic link function. The ‘catch‐curve’ method was applied using MCMC sampling in JAGS, again with the aid of the R2jags package for R.

Only adults were included due to the assumption of constant survival rate between age classes, which is unlikely to hold for metamorphs and YOTY (Wells, 2010). A null model including only an effect of ‘sex’ was first fitted to the data, with the assumption that survival rate of males will be lower, as is commonly observed in anurans. The alternate model was guided by the age structure analysis, including additional effects of wetland hydroperiod and seasonal average air temperature. Missing values of seasonal average air temperature were set to the average air temperature for the season at that site. A climate‐based random effect, allowing for systematic variation in survival rate between semi‐arid and temperate sites, was excluded, as models including this term failed to converge. Models were compared using the Deviance Information Criterion (DIC), and annual survival rates calculated for both sexes. Parameter estimates were obtained from 30,000 MCMC samples after a burn‐in of 30,000 samples.

## 3 RESULTS

### 3.1 Age distribution

**FIGURE 3:**
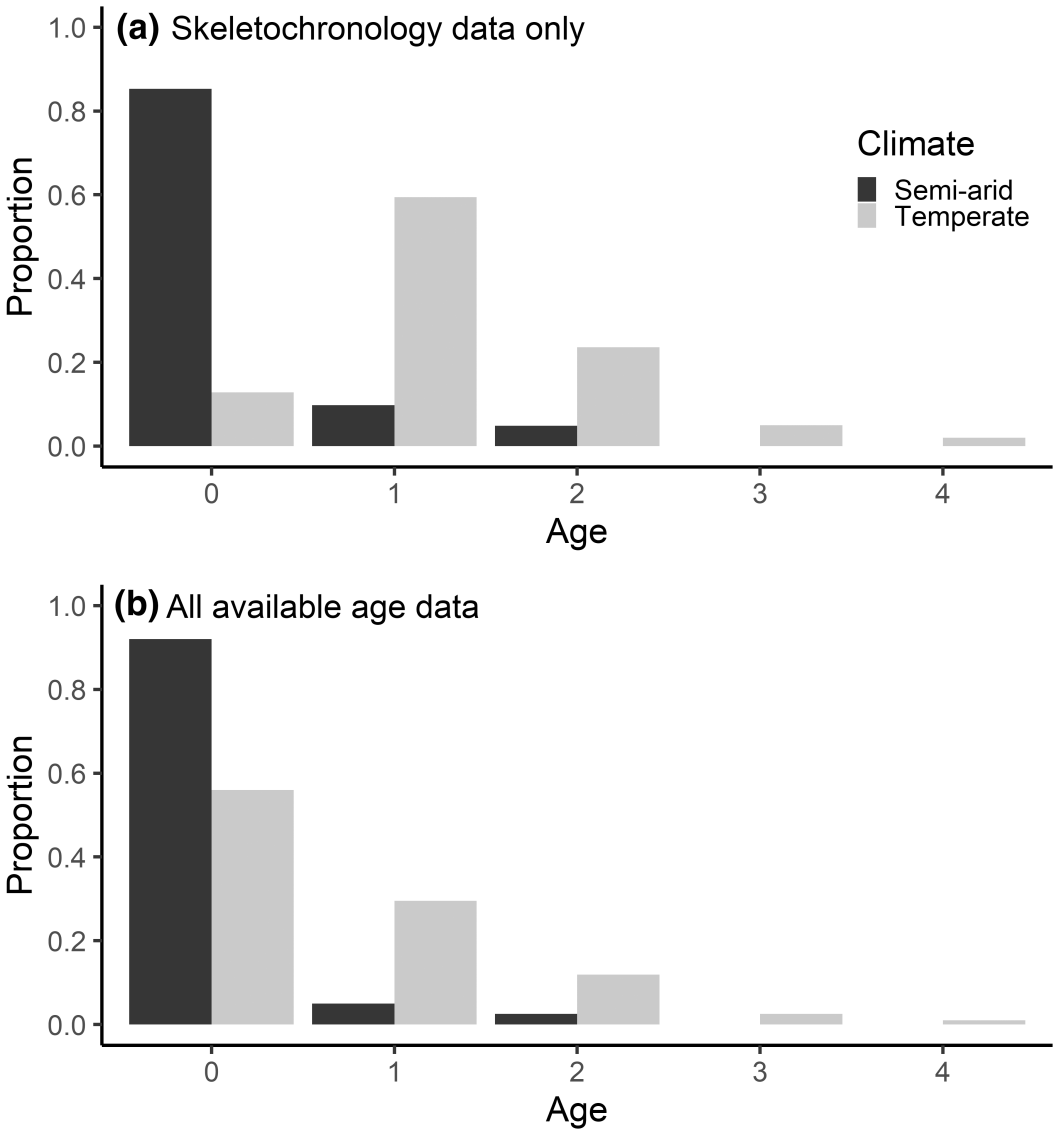
Proportion of *Litoria raniformis* in each age group sampled across the two climatic regions, either based only on ages determined by skeletochronology (a) or including all young‐of‐the‐year captured during surveys (b).

In the semi‐arid region, ages ranged from zero to two years old for *L. raniformis*, and 92% of those captured were young‐of‐the‐year (YOTY). In temperate sites, ages ranged from 0 to 4 years old, with a median age of 1 year. The semi‐arid sites had a substantially higher proportion of YOTY (0.92) than temperate sites (0.55) (Figure 3b). In the temperate region, there was a higher proportion of one and two‐year‐old frogs (0.29 and 0.12, respectively) than the semi‐arid region (0.05 and 0.03 respectively). In the semi‐arid region, there were no frogs detected over the age of two, whereas in the temperate region 15 three‐year‐old and six four‐year‐old frogs were identified, making up 2.5% and 1.0%, respectively of those sampled.

### 3.2 Drivers of age in *Litoria raniformis*

Hydroperiod appeared in the top three models of the drivers of age in *L. raniformis* across the two climatic regions (Table 2). The top model also included seasonal average air temperature, while the second‐best included proportion of water temperature between 17 and 26°C.

**TABLE 2:** Ranking of the top five models describing environmental correlates of age of *Litoria raniformis* across the Lowbidgee, Gippsland, and Melbourne regions. All models include ‘site’ as a random effect. The number of parameters (K), the log likelihood (LogLik), Akaike's information criterion (AIC), distance from the most parsimonious model (ΔAIC), and model selection weight (W) are provided.

Model	Variables	*K*	LogLik	AIC	ΔAIC	*W*
19	Hydroperiod + Season air temperature average	5	−567.80	1145.69	0	0.9
18	Hydroperiod + Season water temperature proportion 17–26°C	5	−569.74	1149.57	3.87	0.1
6	Hydroperiod	4	−574.85	1157.75	12.05	<0.01
3	pH + pH ^2^	5	−575.53	1161.15	15.45	<0.01
13	pH + Season air temperature average	5	−575.92	1161.93	16.23	<0.01

Hydroperiod was positively correlated with age (model coefficient = 1.32, *p* = <.01) indicating a greater frequency of older individuals with increasing water permanence. A negative relationship between age and seasonal average air temperature was also evident (model coefficient = −1.44, *p* = <.01). Similarly, a negative relationship was detected between age and proportion of seasonal water temperature between 17 and 26°C (model coefficient = −1.13, *p* = <.01) (Table 3).The climate‐based random effect could not be estimated for the first two models, being correlated with the seasonal air temperature variables included in these models. However, estimates from the third top model, including an effect of hydroperiod only, confirmed a markedly younger average age for *L. raniformis* in the semi‐arid Lowbidgee relative to the temperate sites sampled. The intercept for semi‐arid sites was −1.85 compared with −0.52 for temperate sites.

**TABLE 3:** Coefficient estimates for each environmental variable identified in the top three models determining age of *Litoria raniformis*. Estimates derive from the top model in which they appeared, with the exception of variables with a quadratic effect, in which estimates derive from the top model including the quadratic effect.

Variable	Estimate	Std. error	*z*‐value	*p*‐value
Intercept	−0.89	0.14	−6.59	<.01
Hydroperiod	1.32	0.26	5.07	<.01
Season air temperature average	−1.44	0.32	−4.48	<.01
Season water temperature proportion 17–26°C	−1.13	0.29	−3.81	<.01

### 3.3 Growth curves

The largest male *L. raniformis* captured in the semi‐arid region was a two‐year old with SUL of 79 mm. In the temperate region, the largest male captured, a four‐year‐old, measured 71 mm. The largest females captured in the temperate region included two four‐year‐olds measuring 97 and 92 mm. As sexing is based on secondary sexual characteristics of the male, it was not possible to sex individuals under 53 mm (the smallest SUL in which male secondary sexual characteristics were detected). Juveniles were therefore those lacking secondary sexual characteristics with SUL <53 mm. The smallest juvenile captured was 33.5 mm.

**FIGURE 4:**
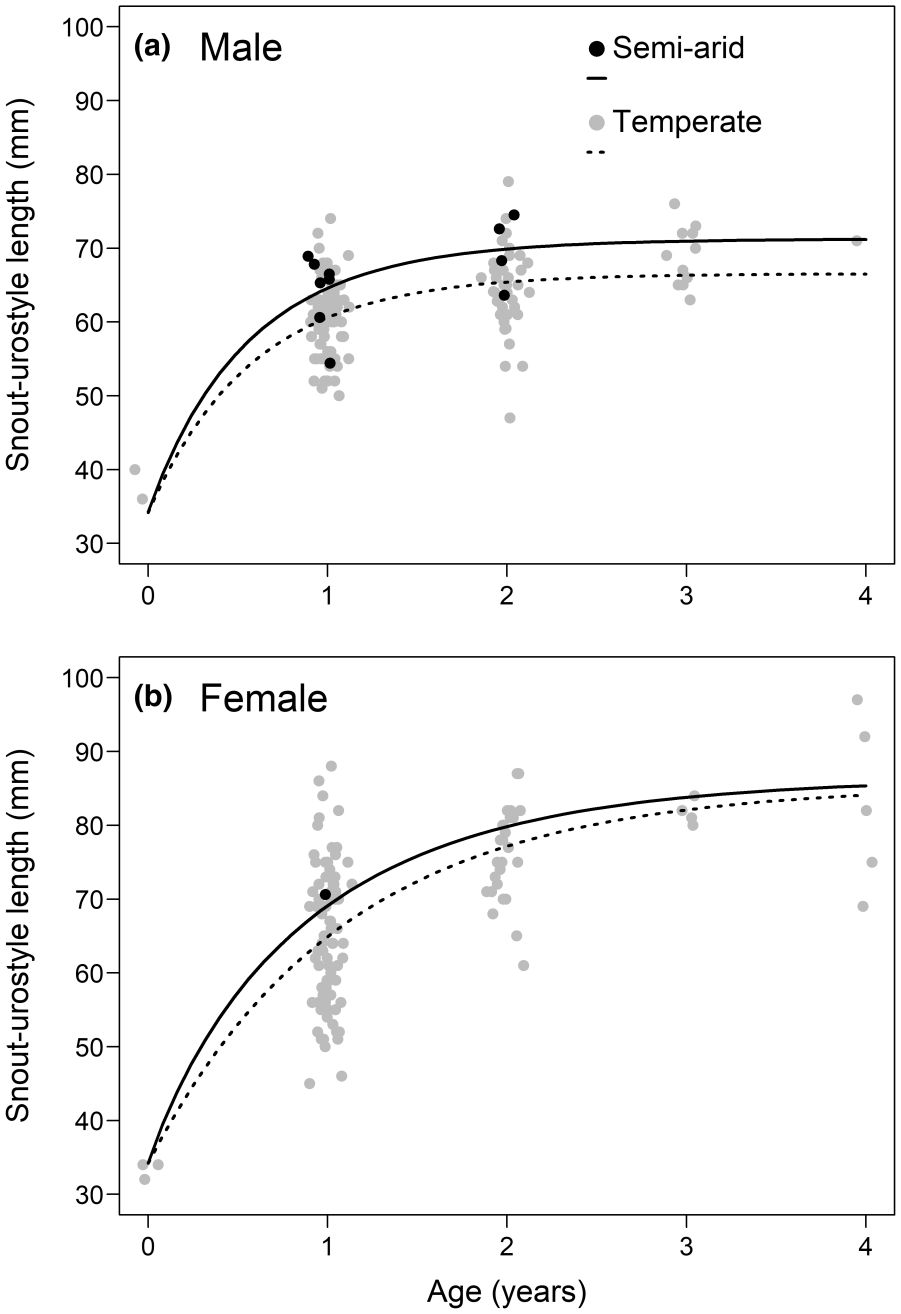
Post metamorphic growth curves for male (a) and female (b) *Litoria raniformis* across two climatic regions: Temperate and semi‐arid. Curves were derived for each sex by estimating the parameters of the von Bertalanffy growth model. Dots are the underlying size‐at‐age data for frogs from both climatic regions. Dots have been slightly jittered on the *x*‐axis to improve readability. The black line represents the predicted average growth rates for each region

Derived growth curves (Figure 4) reveal that SUL increases rapidly and exponentially with almost all the growth occuring in the first year or shortly thereafter. Estimates of the asymptotic body size, α, were lower for males than females in both the semi‐arid (males = 71.15 mm; females = 86.7 mm) and temperate regions (males 66.56 mm and females 85.68 mm), with non‐overlapping 95% CI's (Table 4). The asymptotic body size yet to be attained at birth (β) was identical for females in both climates at 0.60; however, there was a slight difference between the regions for males (β: semi‐arid = 0.52; temperate = 0.49). Estimates of the growth parameter (λ) indicate growth rates are slightly faster in the semi‐arid region (males = 1.95; females = 1.74) than in the temperate region (males: semi‐arid = 1.95, temperate = 1.71; females: semi‐arid = 1.74, temperate = 0.91; Table 4); however, 95% CIs overlapped in all cases.

**TABLE 4:** Estimates of the parameters of the von Bertalanffy growth model for male and female *Litoria raniformis* in the semi‐arid and temperate regions. Parameters are: *α*—The asymptotic snout‐vent length (mm); *β*—The fraction of the asymptotic body size yet to be attained at birth; and *λ*—The growth constant. The 95% confidence interval (95% CI) is provided for each parameter.

Sex	Climate	*α* (95% CI)	*β* (95% CI)	*λ* (95% CI)
Male	Semi‐arid	71.15 (66.05–76.62)	0.52 (0.48–0.55)	1.95 (1.15–5.29)
	Temperate	66.56 (64.59–68.78)	0.49 (0.47–0.50)	1.71 (1.36–2.16)
Female	Semi‐arid	86.7 (77.37–96.10)	0.60 (0.56–0.64)	1.74 (0.61–7.56)
	Temperate	85.68 (80.32–88.5)	0.60 (0.57–0.61)	0.91 (0.79–1.14)

### 3.4 Survival rates

Two model structures were developed for annual survival rates of *L. raniformis* across the two climatic regions: a null model in which only an effect of ‘sex’ was included, and an alternate model including effects of wetland hydroperiod and seasonal average air temperature (guided by their effects on age structure). Support for the null and alternate model was nearly identical (DIC of 5476.80 and 5477.32, respectively). There is evidence of a slightly higher survival rate for males in both the null and alternate models; however, 95% confidence intervals overlap considerably (Table 5). Survival rate showed a weak positive correlation with increasing water permanence (mean estimate = 0.23, 95% CI = −0.66–1.12: Table 6). There was essentially no effect of seasonal average air temperatures on survival rate (estimate = −0.09, 95% CI = −1.35–1.16) (Table 6).

**TABLE 5:** Mean survival rates of male and female *Litoria raniformis* from the null model (sex only) and for each hydroperiod classification (model with hydroperiod).

Sex	Model	Mean survival rate	SD	95% CI
Male	Null	0.31	0.04	0.23–0.39
	Ephemeral	0.27	0.08	0.13–0.46
	Semi‐permanent	0.30	0.05	0.20–0.40
	Permanent	0.33	0.06	0.22–0.45
Female	Null	0.27	0.04	0.18–0.36
	Ephemeral	0.23	0.08	0.10–0.41
	Semi‐permanent	0.25	0.05	0.16–0.36
	Permanent	0.28	0.06	0.17–0.39

**TABLE 6:** Parameter estimates for the effects of sex, hydroperiod, and seasonal average air temperature on adult survival rate of *Litoria raniformis*.

Variable	Parameter estimate	SD	95% CI
Sex	0.24	0.24	−0.22–0.72
Hydroperiod	0.23	0.45	−0.66–1.12
Seasonal average air temperature	−0.09	0.64	−1.35–1.16

## 4 DISCUSSION

Populations of *L. raniformis* in both the semi‐arid and temperate regions were dominated by 1 to 2‐year olds. The skew of the age structure was more pronounced in the semi‐arid region, with 92% of those sampled being young of year (Figure 2b). In line with the effects of *Bd* on age structure in the congener *Litoria verreauxii alpina* (Scheele et al., 2016), we expected a greater skewing of age structure in the temperate region due to higher prevalence of *Bd* (Turner, Wassens, & Heard, 2021) and the continuing effect of *Bd* on survival rates of *L. raniformis* (Heard et al., 2014). However, this was not observed. Instead, it is apparent that specific environmental factors are predominant in determining the variation in age structure between the climatic regions sampled here.

The numerical dominance of younger individuals in semi‐arid regions could be a result of the difference in the dynamics of the habitat in this region. The temperate system in this study had a greater number of permanent sites compared with the floodplain wetlands of the Lowbidgee. These permanent sites are suitable refuges for populations of *L. raniformis* during dry periods enabling them to persist. Similar higher rates of detection of four‐ and five‐year‐old *L. raniformis* were reported in agricultural habitats in the semi‐arid Coleambally Irrigation region where permanent dams and canals are common (Mann et al., 2010). *L. raniformis* and other frog species inhabiting this irrigation area have access to extensive permanent water channels enabling them to survive to ages reflective of those in the temperate regions (Ashworth, 1998; Heard et al., 2012). Despite having similar climates, habitat availability in the Lowbidgee is more dynamic with high annual variability in the availability of breeding habitats and fewer persistent refuges. This corresponds with the positive effect of hydroperiod on age structure (Table 3) detected in this study.

Growth rates were estimated for males and females in both climatic regions. However, the very small sample size for females in the semi‐arid region (*n* = 1) does not allow for firm conclusions. The growth curves (Figure 4) indicate that almost all growth occurs within 200 days of metamorphosis for both male and female *L. raniformis*, consistent with mark‐recapture data by Heard et al. (2012). Regional differences in growth rates were observed with frogs in the semi‐arid region growing slightly faster and to a marginally larger body size for both males and females than those in the temperate region (Figure 4). However, larger female sample size would need to be obtained from the semi‐arid region to verify this difference. Life‐history theory predicts that increased adult mortality can result in earlier maturation (Stearns, 2000). Prior studies have shown that increased adult mortality in fish and mammals caused by disease has resulted in earlier maturity (Jones et al., 2008; Ohlberger et al., 2011; Scheele et al., 2016). By reaching a larger body‐size more rapidly, it could be possible that *L. raniformis* in the semi‐arid region are reproducing with a larger clutch volume earlier in their life than those in the temperate region, a possible compensation for higher rates of adult mortality (Scheele et al., 2016). However, the small differences in growth rates between the climatic regions along with the small sample size in the Lowbidgee do not allow for firm conclusions. A larger sample size would be required to confirm this life‐history theory.

Survival rates could not be compared between climatic regions, as models including a climate‐based effect failed to converge. Therefore, an overall survival rate of males and females across both climatic regions was estimated, with hydroperiod and seasonal average air temperature as additive effects based on the results of the age structure analysis. Annual survival rates were found to be marginally higher for males (0.31) than females (0.27) across both climatic regions and were slightly higher at permanent sites (Table 5). There was a weak effect of sex (estimate = 0.24, 95% CI = −0.22–0.72) and hydroperiod (estimate = 0.23, 95% CI = −0.66–1.12) on adult survival rates. Compared with the mark‐recapture data of Heard et al. (2012), which suggested *L. raniformis* display very low annual rates of survival (estimated at ~0.03), the estimates produced here are considerably higher, at 0.31 for males and 0.27 for females (Table 5). As such, the skeletochronology data acquired in this study support the supposition that estimates of survival of Heard et al. (2012) were lower than expected due to emigration. The survival rate determined in this study was comparable to that detected in the sister species, *Litoria aurea*; Pickett et al. (2014) calculated a mean annual adult survival probability of 0.217 (SD = 0.087). Furthermore, Pickett et al. (2014) found males to have a higher survival rate than females.

It was hypothesized that age structure and survival rates would differ between regions and across gradients of local temperature regimes, pH, and salinity, given the effects of these variables on the prevalence and intensity of *Bd* infections in *L. raniformis* (Turner, Wassens, & Heard, 2021). Little evidence was found to support these hypotheses. Regional differences were suggested by the effect of average seasonal air temperature on age structure (largely a proxy for climatic differences); however, the negative effect was opposite to that hypothesized based on the supposed higher survival rates with warmer temperatures due to lower *Bd* infection risk. Age structure was more positively skewed in the semi‐arid region. Localized factors may therefore have a higher effect on survival of *L. raniformis* than *Bd* alone, diffusing or over‐riding the environmental relationships anticipated from *Bd* dynamics. Such factors include hydroperiod and flooding regime (which received some support in this study), along with the physiological stresses of the climate itself. *L. raniformis* is at the limit of its range in the semi‐arid region surveyed during this study, experiencing greater extremes of temperature and evaporative water loss than temperate region populations. Hence, despite lower *Bd* prevalence and infection intensity, physiological stressors and other environmental factors may have dominant effects on survival rates for *L. raniformis* in semi‐arid environments.

Despite the higher prevalence and intensity of *Bd* in the temperate region (Turner, Wassens, & Heard, 2021), the overall environmental conditions appear to confer higher annual survival rates for *L. raniformis*. The detection of three‐ and four‐year‐olds in the temperate population ensures that at least some individuals survive to reproduce in multiple seasons, conferring populations with lower susceptibility to environmental stochasticity, particularly the resulting in recruitment failure. Nevertheless, local population extinction and re‐colonization is commonplace in the temperate environments studied here (Heard et al., 2012, 2013, 2015). Hence, while the greater longevity of *L. raniformis* in temperature regions indicated by this study is suggestive of greater population stability than in semi‐arid landscapes, they remain fundamentally unstable and reliant on metapopulation processes for persistence.

It is important to acknowledge that longer‐term data may be necessary to ascertain the true demographic differences across the range of *L. raniformis*. Sampling occurred over only one season in the semi‐arid region, and over two seasons for most sites sampled in the temperate region (Mann et al., 2010; Scheele et al., 2015). Consequently, the age structures observed may not be characteristic of these populations, particularly if they display considerable variation from year‐to‐year (Heard et al., 2012; Muths et al., 2011). A larger sample size from both temperate and semi‐arid region along with repeat sampling over a number of years (4–10) would enable stronger conclusions.

Age structure, growth, and survival rates are vital demographic parameters for guiding the conservation management of species at risk of decline or extinction. Estimates of demographic parameters can be used for population modeling and population viability analysis (PVA) (Morris & Doak, 2002), which may in turn be used to identify key life stages as management targets, determine minimum viable populations sizes, determine how many individuals to release into a population for augmentation purposes, or decide how many populations are needed to protect a species from regional or global extinction (Morris & Doak, 2002). Furthermore, PVA's can be used to project the likely impact of stochastic events such as disease, fire, drought, or wetland inundation frequencies on populations at risk (Mathwin et al., 2021; Potvin et al., 2017). For species such as *L. raniformis* in the Lowbidgee wetlands, where inundation frequency, extent and duration are heavily managed, using survival rates to inform PVA's could lead to management actions which ensure long‐term survival. Factors such as the timing of environmental watering, the resultant water temperature, and the impact on chytrid infection burdens and therefore survival rates could be further explored.

## AUTHOR CONTRIBUTIONS

**Anna Turner:** Conceptualization (equal); data curation (equal); formal analysis (equal); investigation (equal); methodology (equal); project administration (lead); validation (equal); writing – original draft (equal); writing – review and editing (equal). **Geoffrey Heard:** Conceptualization (equal); data curation (equal); formal analysis (equal); funding acquisition (equal); investigation (equal); methodology (equal); software (equal); supervision (equal); validation (equal); writing – review and editing (equal). **Andrew Hall:** Writing – review and editing (equal). **Skye Wassens:** Conceptualization (equal); funding acquisition (equal); methodology (equal); supervision (equal); validation (equal); writing – review and editing (equal).

## CONFLICT OF INTEREST

The authors declare that they have no conflict of interest.

## FUNDING INFORMATION

Fieldwork in Gippsland and skeletochronology of sample collected was funded by Greening Australia facilitated through Martin Potts. Fieldwork and skeletochronology in the Lowbidgee region was funded by Australian Research and Training Program (ARTP) Scholarship and top‐up scholarship from Institute of Land, Water and Society (ILWS), Charles Sturt University. Fieldwork and processing of toe clips from Melbourne was funded by the Growling Grass Frog Trust Fund, La Trobe University and Victorian Department of Environment, Land, Water and Planning. AT was supported by an ARTP Scholarship and top‐up scholarship from ILWS, Charles Sturt University.

## Supporting information


**TABLE S1** Hypothesized effects of environmental variables on the age of *Litoria raniformis* across semi‐arid and temperate regions. Arrows indicate the direction of the proposed relationship with the corresponding model structure provided. All models include site as a random effect.

## Data Availability

The dataset generated during and analysed during the current study are published open access and available from https://doi.org/10.5281/zenodo.5879959.
