# Supplementary material for: Age structure of amphibian populations with endemic chytridiomycosis, across climatic regions with markedly different infection risk
Source: Ecol Evol. 2022 Jul 24;12(7):e9123. doi: 10.1002/ece3.9123 (PMC9309026; doi:10.1002/ece3.9123)
Supplement: Supplementary file 1 — TABLE S1 Hypothesized effects of environmental variables on the age of Litoria raniformis across semi‐arid and temperate regions. Arrows indicate the direction of the proposed relationship with the corresponding model structure provided. All models include site as a random effect. [file ECE3-12-e9123-s001.docx]

Supplementary Material:

Age structure of amphibian populations with endemic chytridiomycosis, across climatic regions with markedly different infection risk

Oecologia

Anna Turner^A,D^, Geoffrey Heard^B^, Andrew Hall ^A,B^ and Skye Wassens^A,B^

^A^ School of Agricultural, Environmental and Veterinary Science, Charles Sturt University, Albury Wodonga Campus, PO Box 789, Albury, NSW, 2640 Australia;

^B^ Institute of Land, Water and Society, Charles Sturt University, Albury-Wodonga Campus, PO Box 789, Albury, NSW, 2640 Australia;

^D^ Corresponding author (email: [aturner@csu.edu.au](mailto:aturner@csu.edu.au))

**Table S1**. Hypothesised effects of environmental variables on the age of Litoria raniformis across semi-arid and temperate regions. Arrows indicate the direction of the proposed relationship with the corresponding model structure provided. All models include site as a random effect.

| Candidate model | Hypothesis | Corresponding model |
| --- | --- | --- |
| 1 | Null hypothesis | - |
| 2 | ↑ *Bd* prevalence with ↑ in pH, ↓ age | pH |
| 3 | ↑ *Bd* prevalence with ↑ in pH and ↓ with ↑ pH2, ↓ age | pH + pH² |
| 4 | ↓ in salinity , ↑ *Bd* prevalence, therefore ↓ age | Salinity |
| 5 | ↑ emergent vegetation, ↑ *Bd* prevalence, ↓ age | Emergent vegetation |
| 6 | ↑ in age with ↑ in hydroperiod | Hydroperiod |
| 7 | ↓ age with ↑ in proportion night time air temperature between 17-26^◦^C | Season air temperature proportion 17-26^◦^C |
| 8 | ↑ age with ↓ in average night time air temperature | Season air temperature average |
| 9 | ↓ age with ↑ in proportion night time water temperature between 17-26^◦^C | Season water temperature proportion 17-26^◦^C |
| 10 | ↑ age with ↓ in average night time water temperature | Season water temperature average |
| 11 | ↑ in pH, ↑ in proportion night time water temperature between 17-26^◦^C, ↓ age | pH + Season air temperature proportion 17-26^◦^C |
| 12 | ↑ in pH and ↓ with ↑ pH2, ↑ in proportion night time water temperature between 17-26^◦^C, ↓ age | pH + pH² + Season air temperature proportion 17-26^◦^C |
| 13 | ↑ in pH, ↑ in average night time air temperature, ↓ age | pH + Season air temperature average |
| 14 | ↑ in pH, ↑ average night time air temperature, ↓ age | pH + pH² + Season air temperature average |
| 15 | ↓ in salinity , ↑ in proportion night time water temperature between 17-26^◦^C, ↓ age | Salinity + Season air temperature proportion 17-26^◦^C |
| 16 | ↓ in salinity , ↑ in average night time air temperature, ↓ age | Salinity + Season air temperature average |
| 17 | ↓ in salinity , ↑ in average night time water temperature, ↓ age | Salinity + Season water temperature average |
| 18 | ↓ in hydroperiod, ↑ in proportion night time air temperature between 17-26^◦^C, ↓ in age | Hydroperiod + Season air temperature proportion 17-26^◦^C |
| 19 | ↓ in hydroperiod , ↑ in average night time air temperature, ↓ age | Hydroperiod + Season air temperature average |
